# Electrophysiological activity pattern of mouse hippocampal CA1 and dentate gyrus under isoflurane anesthesia

**DOI:** 10.3389/fncel.2024.1392498

**Published:** 2024-07-17

**Authors:** Rui Wang, Linzhong Zhang, Xia Wang, Wen Li, Tingliang Jian, Pengcheng Yin, Xinzhi Wang, Qianwei Chen, Xiaowei Chen, Han Qin

**Affiliations:** ^1^Department of Anesthesiology, Shanxi Medical University and Second Hospital of Shanxi Medical University, Taiyuan, China; ^2^Guangyang Bay Laboratory, Chongqing Institute for Brain and Intelligence, Chongqing, China; ^3^Center for Neurointelligence, School of Medicine, Chongqing University, Chongqing, China; ^4^Brain Research Center and State Key Laboratory of Trauma and Chemical Poisoning, Third Military Medical University, Chongqing, China; ^5^Department of Rehabilitation Medicine, Xijing Hospital, Fourth Military Medical University, Xi’an, China

**Keywords:** general anesthesia, CA1, dentate gyrus, electrophysiological recording, isoflurane

## Abstract

General anesthesia can impact a patient’s memory and cognition by influencing hippocampal function. The CA1 and dentate gyrus (DG), serving as the primary efferent and gateway of the hippocampal trisynaptic circuit facilitating cognitive learning and memory functions, exhibit significant differences in cellular composition, molecular makeup, and responses to various stimuli. However, the effects of isoflurane-induced general anesthesia on CA1 and DG neuronal activity in mice are not well understood. In this study, utilizing electrophysiological recordings, we examined neuronal population dynamics and single-unit activity (SUA) of CA1 and DG in freely behaving mice during natural sleep and general anesthesia. Our findings reveal that isoflurane anesthesia shifts local field potential (LFP) to delta frequency and reduces the firing rate of SUA in both CA1 and DG, compared to wakefulness. Additionally, the firing rates of DG neurons are significantly lower than CA1 neurons during isoflurane anesthesia, and the recovery of theta power is slower in DG than in CA1 during the transition from anesthesia to wakefulness, indicating a stronger and more prolonged impact of isoflurane anesthesia on DG. This work presents a suitable approach for studying brain activities during general anesthesia and provides evidence for distinct effects of isoflurane anesthesia on hippocampal subregions.

## 1 Introduction

General anesthesia, induced by a diverse array of drugs, results in reversible loss of consciousness, enabling patients to safely undergo surgical procedures (Urban and Bleckwenn, 2002; Alkire et al., 2008; Franks, 2008; Akeju and Brown, 2017). Although general anesthesia shares some similarities with sleep in terms of electroencephalogram (EEG) patterns and decreased arousal state, it diverges notably when considering their impact on memory and cognitive functions. Sleep is crucial for memory consolidation (Brown et al., 2010; Diekelmann and Born, 2010; Girardeau and Lopes-Dos-Santos, 2021), while general anesthesia may affect postoperative memory and cognitive function, particularly in the elderly and infants (Moller et al., 1998; Alam et al., 2018). Numerous studies have established a correlation between postoperative cognitive dysfunction and the effects of anesthetics on the hippocampus (Feng et al., 2017; Yang et al., 2021).

The hippocampus, a pivotal node for cognition, comprises several subregions, including the dentate gyrus (DG), CA1, CA2, and CA3. Among the most studied information routes in the hippocampus is the trisynaptic circuit, with the initial synaptic junctions in DG and the final ones in CA1 (Knowles, 1992). This circuit is vital for memory and navigation (Nakashiba et al., 2009). Although the structures and functions of CA1 and DG closely interconnect, significant distinctions exist between them. The CA1 region consists of compact, broad-based pyramidal cell, while the DG area is characterized by comparably dense but notably smaller granule cell (Stuart and Spruston, 1998). Furthermore, variations in receptor types (Song et al., 2001), synaptic plasticity (Kramár et al., 2012), and responses to external stimuli (Alkadhi, 2019) are evident in both CA1 and DG. Notably, CA1 and DG demonstrate diverse reactions to ischemia (Hsu et al., 1998), stress (Gerges et al., 2004), and Alzheimer’s disease (Dao et al., 2013). Anesthesia-related findings propose that midazolam is more effective in reducing the amplitude of intracellular action potentials and excitatory postsynaptic potential slopes in DG granule cells than in CA1 pyramidal cells (Sato et al., 1997; Kobayashi et al., 2004). Additionally, CA1 and DG exhibit differential responses to sevoflurane anesthetic (Hirota and Roth, 1997).

Despite these identified distinctions, a comprehensive study regarding the specific influence of isoflurane anesthesia on CA1 and DG neuronal activities, at both population and single-cell levels in freely behaving animals, remains insufficiently explored. To address this issue, a proper recording approach is required. In previous investigations, *in vitro* electrophysiology (Simon et al., 2001; Li et al., 2022), fiber photometry (Bao et al., 2021), and two-photon microscopy (Bharioke et al., 2022) have been employed to monitor neuronal activity under anesthesia. However, limitations persist within these methods. *In vitro* electrophysiological recordings permit the monitoring of single-cell activity but are confined to brain slices (Yang et al., 2021; Yan et al., 2023). Fiber photometry offers an effective means of observing population Ca^2+^ activity in freely behaving animals but lacks single-cell resolution (Bao et al., 2021; Wang Z. Q. et al., 2023). Two-photon microscopy is suitable for Ca^2+^ recordings in anesthetized or head-fixed animals with high spatial resolution, yet the recording regions are primarily restricted to superficial areas (Denk et al., 1990; Yang et al., 2021). *In vivo* electrophysiology emerges as a robust technique for investigating the local field potential (LFP) and single-unit activity (SUA) of deep brain regions in freely behaving animals (Kuang et al., 2010; Yang et al., 2021). This approach provides a suitable method for recording neuronal activities throughout the general anesthesia process.

In this study, we amalgamated *in vivo* electrophysiological recordings with EEG-electromyogram (EMG) and behavioral video recordings to investigate neuronal activities in CA1 and DG regions during natural sleep and isoflurane-induced general anesthesia in freely behaving mice. We identified a shift toward lower frequency bands in the LFP spectrogram and a decrease in SUA during isoflurane anesthesia in both CA1 and DG. Furthermore, the LFP spectrogram recovered earlier in CA1 than in DG from anesthesia. Additionally, the firing rates of CA1 neurons were higher than those of DG neurons during anesthesia. These results establish a robust approach for investigating neuronal activities during general anesthesia and reveal distinct activity patterns in CA1 and DG, contributing to a better understanding of memory and cognition impairments induced by anesthesia.

## 2 Materials and methods

### 2.1 Mice

We utilized adult male C57BL/6 J mice (3–4 months old) for the electrophysiological surgery in this study. Prior to the surgery, mice were group-housed, and those implanted with tetrodes were individually housed. The mice received adequate food and water under a 12/12 h light/dark cycle (lights at 8:00 a.m.), maintaining a constant environment at 21–24°C with 50–60% relative humidity. All procedures adhered to institutional animal welfare guidelines and were approved by the Third Military Medical University Animal Care and Use Committee.

### 2.2 Tetrodes assembly fabrication

Tetrodes were crafted from 25-μm-diameter insulated tungsten wire (California Fine Wire, CFW2002936) (Yang et al., 2023). In brief, a 30-cm-long tungsten wire was folded twice and twisted using an electromagnetic stirrer (Apera Instruments, 801 Magnetic Stirrer) to form a tetrode. The tetrode was then vertically aligned and securely fused by melting the insulation with a heat gun (450°C, 1 min). A 1.2-cm-long silica capillary tube (Polymicro Technologies, TSP100170; ID, 100 μm; OD, 164 μm) was applied and affixed with super glue to the tetrode tip for protection and support. Four tetrodes were arranged in a line with a 200 μm spacing, connecting the other end of the 16 electrodes (4 × 4 tetrodes) to a 16-channel connector (Omnetics, A79016-001) via gold pins (Neuralynx, small EIB pins).

A microdrive, composed of a metal rod, three plastic blocks, and a screw-nut pair, facilitated vertical movement of the tetrodes. The plastic blocks were threaded onto the metal rod and screw, with two blocks secured to the ends using super glue and the third block positioned in the middle. The previously prepared tetrodes were fastened to the middle block, allowing for vertical movement when the screw was turned.

### 2.3 Stereotaxic surgery

**Figure 1:**
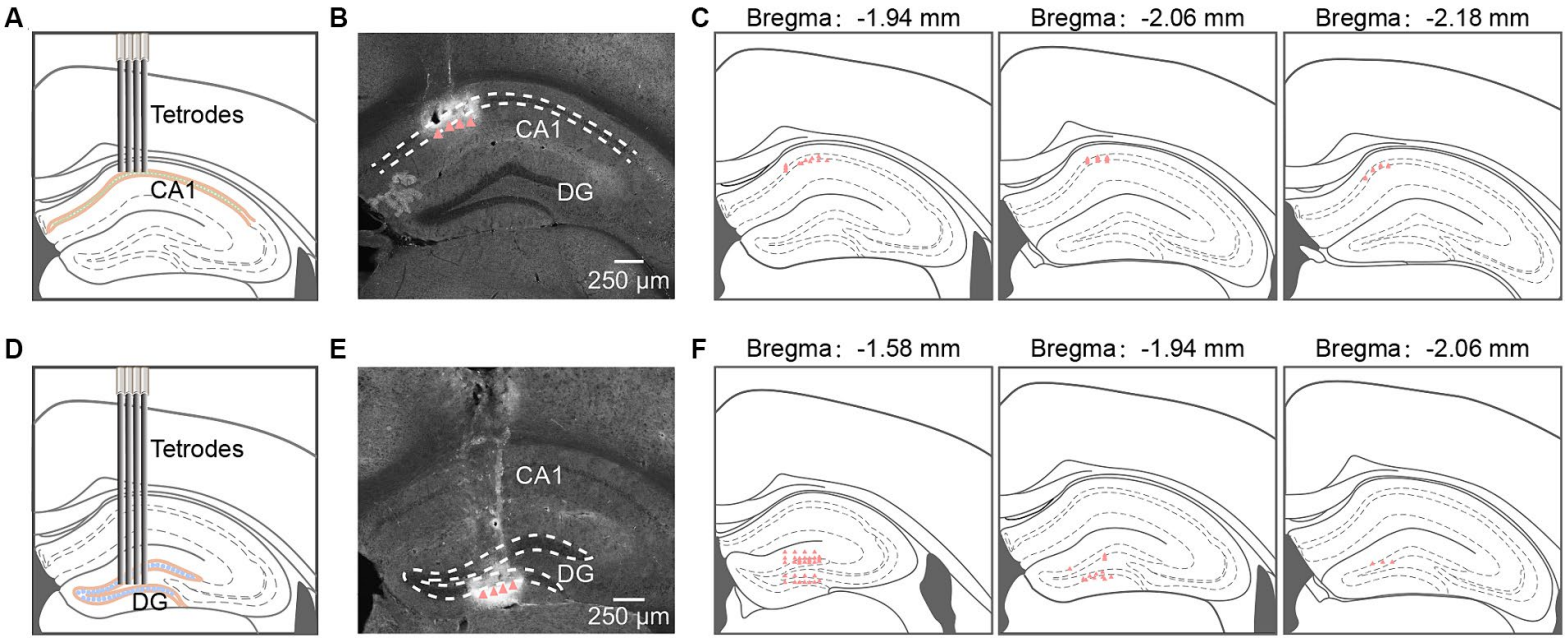
Tetrodes implantation in hippocampal CA1 and DG. **(A,D)** Schematic diagram of tetrodes implantation in CA1 **(A)** and DG **(D)**. **(B,E)** Representative coronal sections showing locations of tetrode tips in CA1 **(B)** and DG **(E)**. Arrowheads identify tetrode tips. **(C,F)** Summary of tetrode locations in CA1 (**C**, *n* = 32 from 4 mice) and DG (F, *n* = 48 from 5 mice). DG, dentate gyrus.

Mice were anesthetized with isoflurane in oxygen (3% induction, maintained at 1–2%, 0.5 L/min). Placed in a stereotactic apparatus with a heating pad maintaining a ~ 37°C temperature, an incision was made to expose the skull at the top of the head after hair removal. A small craniotomy (0.5 × 0.5 mm) was performed above dorsal CA1 (AP: −1.85 mm, ML: 1.10 mm, Figure 1). The prepared tetrodes were inserted through the craniotomy to a depth of 0.9 mm (for CA1 recording) or 1.7 mm (for DG recording). EEG–EMG electrodes were implanted for recording signals. Two stainless screws were inserted into craniotomy holes above the frontal lobe for EEG recording, and two EMG electrodes were inserted into the neck muscles for EMG recording. Dental cement secured the tetrodes and EEG–EMG electrodes to the skull. Post-surgery, mice were returned to warm cages for full recovery and received intraperitoneal injections of dexamethasone sodium phosphate (1 mg/mL, 0.1 mL/10 g/d) and ceftriaxone sodium (50 mg/mL, 0.1 mL/10 g/d) for three consecutive days to minimize inflammation.

### 2.4 *In vivo* electrophysiological recording

**Figure 2:**
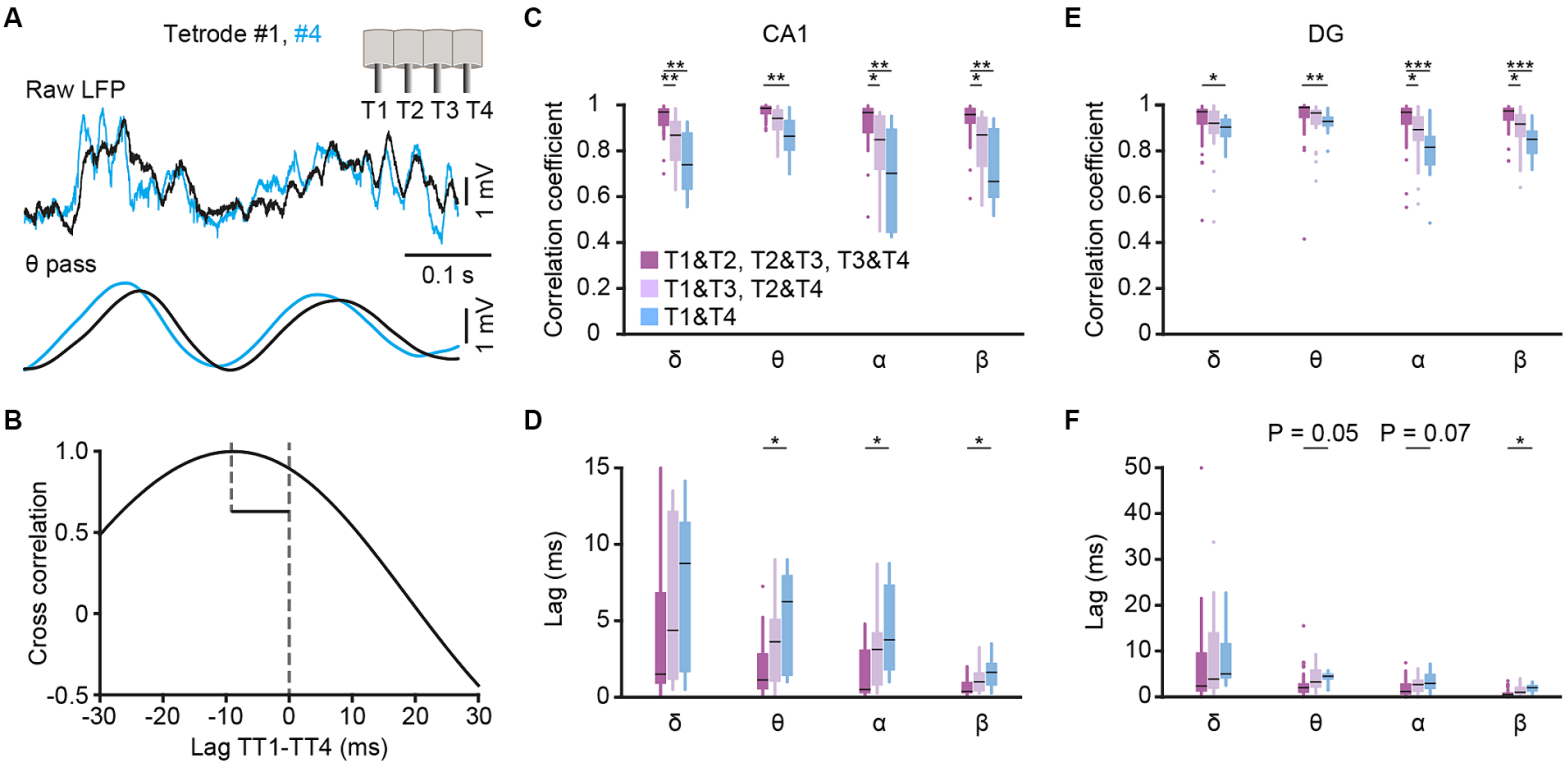
Cross-correlation analysis among different tetrodes. **(A)** Example traces of CA1 LFPs simultaneously recorded from tetrode #1 (black) and #4 (blue). The raw traces (top) and theta filtered traces (bottom, band-pass filtered from 6–12 Hz) are presented, and the insert in the upright corner shows the tetrodes arrangement. **(B)** Lagged cross-correlation of theta filtered LFP between tetrode #1 and #4. **(C–F)** Cross correlation coefficient **(C,E)** and time lag **(D,F)** of δ-, θ-, α- and β-filtered LFP among tetrodes with different distances in CA1 **(C,D)** and DG **(E,F)**. CA1: T1&T2, T2&T3, T3&T4 group, *n* = 24 from 4 mice; T1&T3, T2&T4 group, *n* = 16 from 4 mice; T1&T4 group, *n* = 8 from 4 mice. DG: T1&T2, T2&T3, T3&T4 group, *n* = 36 from 5 mice; T1&T3, T2&T4 group, *n* = 24 from 5 mice; T1&T4 group, *n* = 12 from 5 mice. Kruskal-Wallis 1-way ANOVA with Tukey post-hoc comparison test. Box-and-whisker plot: center line, median; box, 25–75% interquartile range (IQR); the whiskers extend to the most extreme data points that are not outliers. *p < 0.05; **p < 0.01; ***p < 0.001.

**Figure 3:**
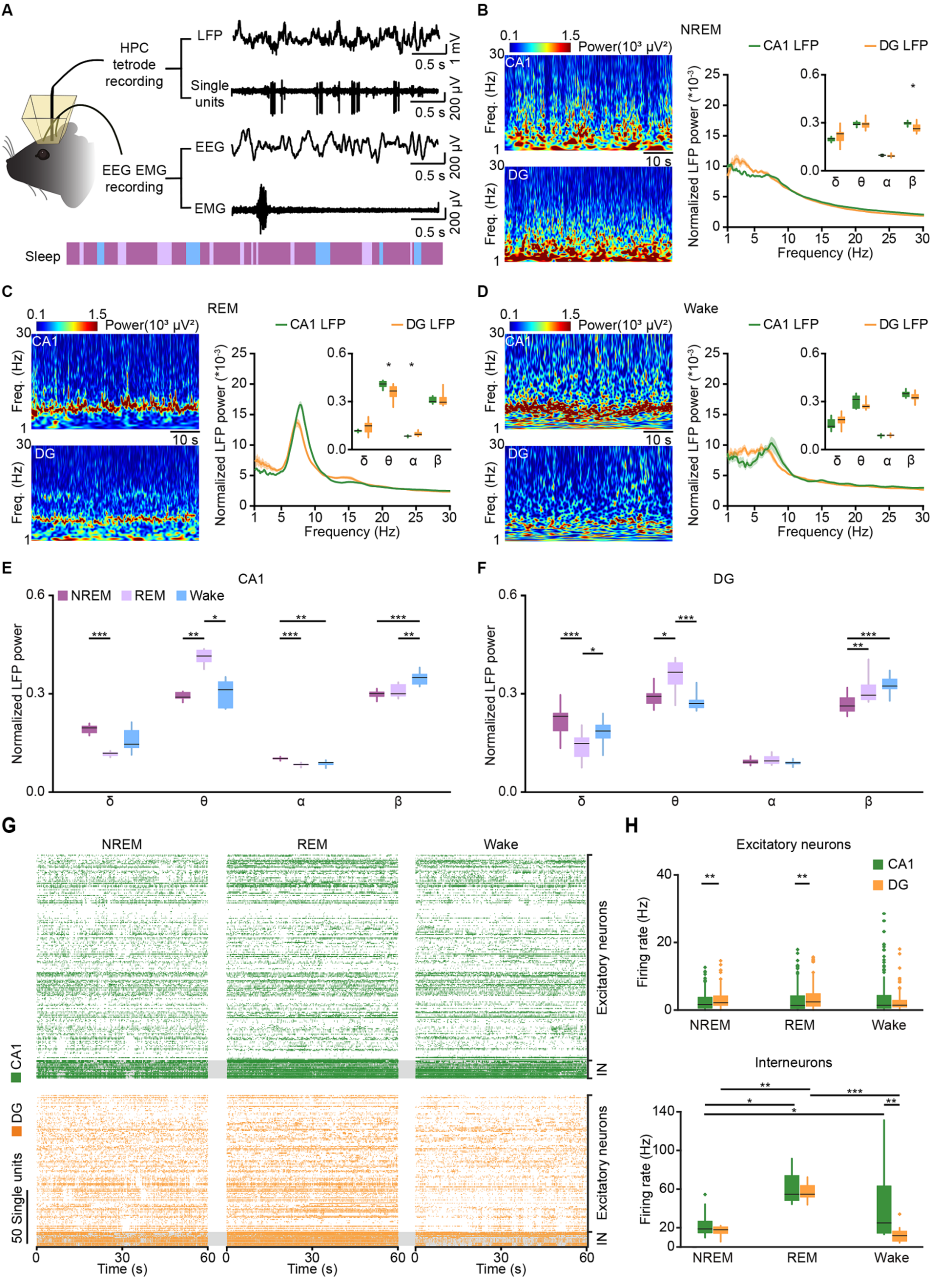
Tetrode recordings in CA1 and DG across sleep-wakefulness cycles. **(A)** Schematic diagram and example traces showing LFP, SUA, and EEG–EMG signals recorded across sleep-wakefulness cycles. **(B–D)** Representative heatmaps (left) and power spectral analyses (right) of CA1 and DG LFPs during NREM sleep **(B)**, REM sleep **(C)**, and wakefulness **(D)**. CA1, *n* = 8 from 4 mice; DG, *n* = 12 from 5 mice, unpaired *t*-test. **(E,F)** Comparison of normalized LFP powers between CA1 **(E)** and DG **(F)** during NREM sleep, REM sleep, and wakefulness. Data from **B–D**. CA1, *n* = 8 from 4 mice; DG, *n* = 12 from 5 mice, RMs 1-way ANOVA with Sidak post-hoc comparison test. **(G)** Raster plots showing the SUA of putative excitatory neurons and putative interneurons in CA1 (orange) and DG (green) during NREM sleep, REM sleep, and wakefulness. **(H)** Average firing rates of putative excitatory neurons (top) and putative interneurons (bottom) in CA1 and DG during NREM sleep, REM sleep, and wakefulness. CA1 excitatory neurons, *n* = 189; DG excitatory neurons, *n* = 125; CA1 interneurons, *n* = 17; DG interneurons, *n* = 13. Friedman’s ANOVA test with Bonferroni post-hoc comparisons. Box-and-whisker plot: center line, median; box, 25–75% interquartile range (IQR); the whiskers extend to the most extreme data points that are not outliers. *p < 0.05; **p < 0.01; ***p < 0.001; LFP, local field potential; SUA, single-unit activity; Freq., frequency; IN, interneuron.

**Figure 4:**
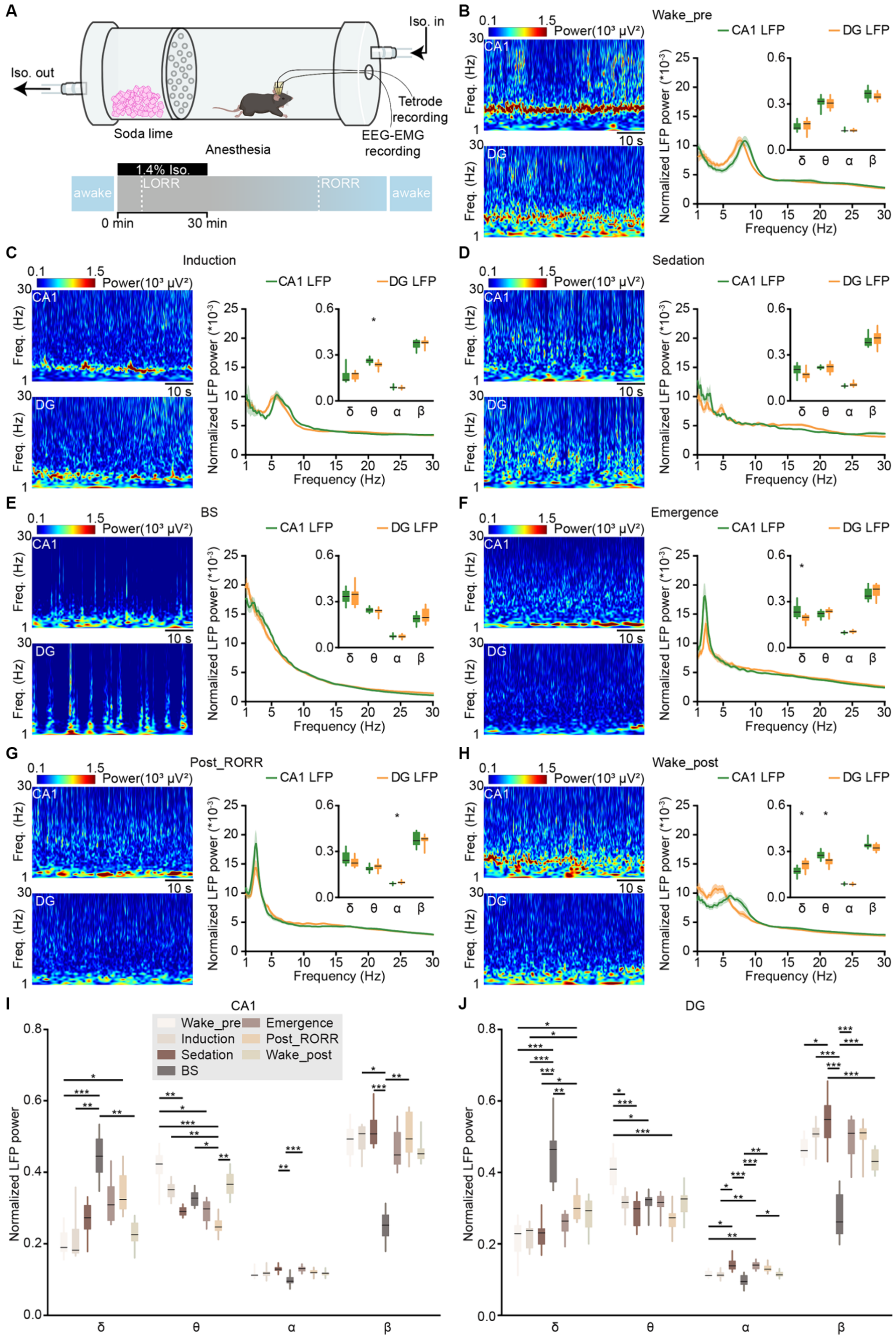
LFPs in CA1 and DG during anesthesia and wakefulness. **(A)** Schematic diagram showing the experimental equipment and protocol for tetrode recordings during isoflurane-induced anesthesia. **(B–H)** Representative heatmaps (left) and power spectral analyses (right) of CA1 and DG LFPs in different anesthesia stages: wake-pre **(B)**, induction **(C)**, sedation **(D)**, BS **(E)**, emergence **(F)**, post_RORR **(G)**, and wake-post **(H)**. CA1, *n* = 8 from 4 mice; DG, *n* = 12 from 5 mice, unpaired *t*-test. **(I,J)** Comparison of normalized LFP powers between CA1 **(I)** and DG **(J)** in different anesthesia stages. Data from **B–H**. CA1, *n* = 8 from 4 mice; DG, *n* = 12 from 5 mice, RMs 1-way ANOVA with Sidak post-hoc comparison test. Box-and-whisker plot: center line, median; box, 25–75% interquartile range (IQR); the whiskers extend to the most extreme data points that are not outliers. *p < 0.05; **p < 0.01; ***p < 0.001. LFP, local field potential; LORR, loss of the righting reflex; RORR, recovery of the righting reflex; Freq., frequency.

Following complete recovery from surgery, as evidenced by an increase in mouse weight, we conducted electrophysiological recordings (Figure 2A). Tetrodes were progressively advanced to a final depth of 1.1 mm for CA1 pyramidal cell recording or 1.85 mm for DG granule cell recording. Recordings were performed during the light phase after mice had been acclimated to the recording cables for 2–3 days. Electrophysiological and EEG–EMG signals were acquired at 20 KHz using an RHD2000 USB board (Intan Technology, C3100) and amplified by a 16-channel digital amplifier (Intan Technology, C3334). Simultaneously, behavioral videos were recorded at 25 Hz. Recordings initially spanned at least 2 sleep-wakefulness cycles (Figure 3A). Subsequently, mice were placed in a chamber for a 5–15-min recording during freely exploring states. Following this, recordings under isoflurane anesthesia were conducted, with mice positioned in a self-made horizontal Plexiglas® cylinder (30 cm long, 15 cm in diameter, Figure 4A). A continuous delivery of 1.4% isoflurane in air (2.5 L/min) was maintained for 30 min. The isoflurane was then discontinued, and only air was delivered continuously. Approximately half an hour after anesthesia recovery, another 5–15-min recording during freely exploring states was performed. Following all recordings, electrical lesions (20 μA for 15 s, DC currents) were applied to verify the recording sites.

### 2.5 Demarcation of general anesthesia

The assessment of anesthetic effects on consciousness relied on the righting reflex test (Solt et al., 2011; Pal et al., 2018) and specific EEG signals (Shander et al., 2018) as identified in previous studies. The anesthetic procedure was categorized into five stages based on these approaches. Isoflurane anesthesia was administered in a horizontal Plexiglas® cylinder, as previously described. During isoflurane delivery, the cylinder was manually rotated 180° every 15 s to place the mice on their backs. The time at which mice lost their ability to right themselves within 30 s was defined as the occurrence of the loss of the righting reflex (LORR). The period from the onset of isoflurane exposure to the occurrence of LORR was termed the induction period (284.3 ± 15.7 s in our dataset). Burst suppression (BS) describes an EEG pattern with a continuous alternating appearance of mixed-frequency activity and inactivity periods (Lewis et al., 2013). The period from the occurrence of LORR to the occurrence of BS was defined as the sedation period (107.4 ± 16.4 s in our dataset). After isoflurane shut off, the BS activity persisted and gradually disappeared. The period from the occurrence of BS activity to its disappearance was termed the BS period. Subsequently, mice regained the ability to right themselves as anesthesia gradually wore off. The period from the disappearance of BS activity to the recovery of the righting reflex (RORR) was defined as the emergence period. Lastly, a 5–10-min period from the occurrence of RORR was labeled as post_RORR. Additionally, wake_pre and wake_post represented the awake state before and after anesthesia.

### 2.6 Histology

48 h post-electrical lesions, mice underwent intraperitoneal injection of pentobarbital for anesthesia, followed by sequential perfusion with saline and 4% paraformaldehyde (PFA) in PBS. The brain was then extracted and immersed in 15% sucrose PBS for overnight dehydration. Subsequently, coronal sections of 50 μm thickness were obtained from the brains. These sections were stained with DAPI (4′,6-diamidino-2-phenylindole, 1,10,000, D9564, Sigma-Aldrich) and imaged using a wide-field fluorescence microscope (Nikon, ECLIPSE Ni) to visualize the recording sites.

### 2.7 Data processing and statistical analysis

#### 2.7.1 Local field potential processing

**Figure 5:**
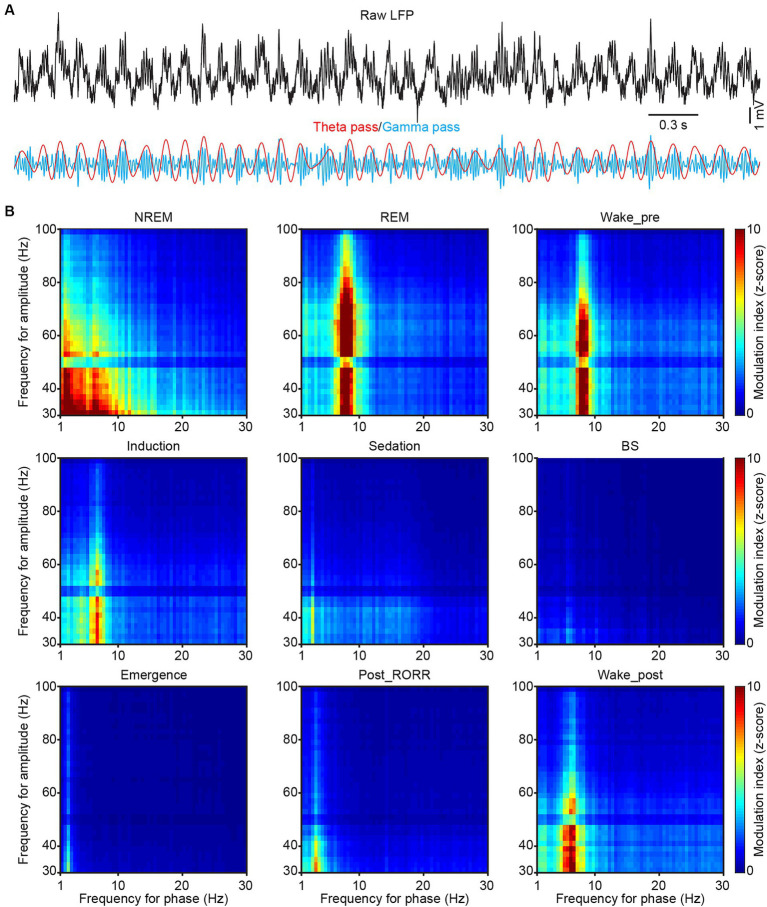
Phase-amplitude coupling in CA1 during sleep and anesthesia. **(A)** Representative raw (black), theta-filtered (red) and gamma-filtered (blue) LFP traces showing the gamma envelope is phase-locked at the peak of the theta oscillation. **(B)** Representative phase-amplitude comodulograms recorded in the CA1 LFP in different sleep and anesthesia stages. RORR, recovery of the righting reflex.

**Figure 6:**
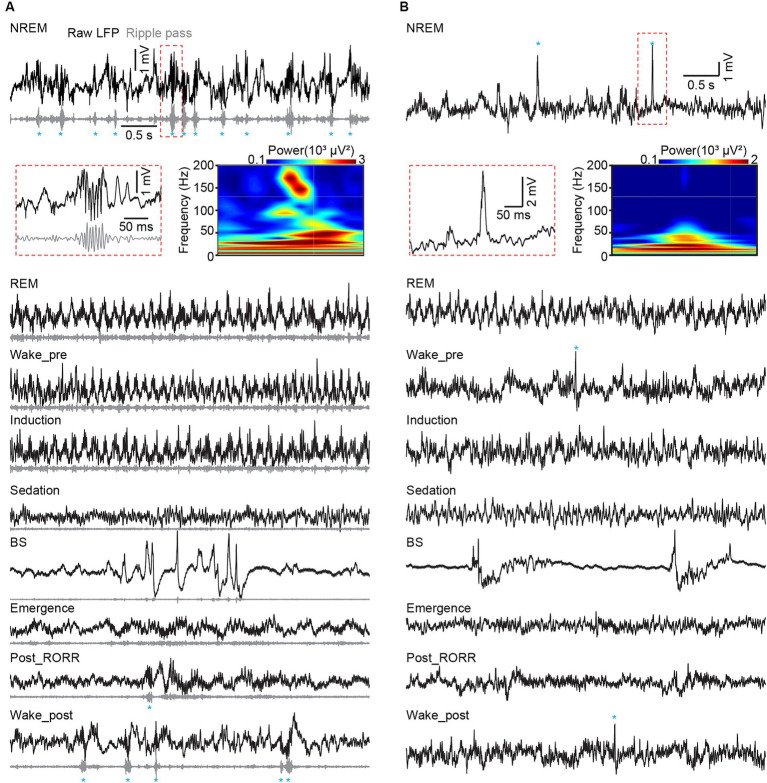
Hippocampal ripples in CA1 and dentate spikes in DG across sleep and anesthesia. **(A)** Representative raw LFP traces (black), rippled band filtered LFP (gray) and ripple events (marked by blue asterisks) recorded in different sleep and anesthesia stages in CA1. **(B)** Raw LFP traces (black) and dentate spike events (marked by blue asterisks) recorded in different sleep and anesthesia stages in DG. Heatmap showing the power spectrogram of a ripple event in CA1 **(A)** and a dentate spike in DG **(B)**. RORR, recovery of the righting reflex.

Spectral profiles of LFP and EEG activities were analyzed using a self-designed MATLAB program. The raw data were bandpass filtered (1 Hz to 30 Hz) for further analysis, with frequency bands consisting of delta (δ: 1–4 Hz), theta (θ: 6–12 Hz), alpha (α: 12–15 Hz), and beta (β: 15–30 Hz) (Boyce et al., 2016; Qin et al., 2022). The raw LFP and EEG data were subjected to Fast Fourier Transform with a frequency resolution of 0.15 Hz. PAC (phase-amplitude coupling) was calculated on 1 to 100 Hz filtered CA1 LFP signal with the PAC toolbox (Yang et al., 2021). The range of phase vector was set as 1–30 Hz, and the range of amplitude vector was set as 30–100 Hz (Figure 5B). Ripples were analyzed in the CA1 LFPs. The raw data were bandpass-filtered within the range of 100–250 Hz, and the events that surpassed 5 SDs above background were marked as ripple events in Figure 6A. Dentate spike analysis was performed in the DG LFPs. Similar to ripple detection, the raw signals were bandpass-filtered between 5 and 100 Hz, and the events that exceeded 5 SDs of the filtered signal were identified as dentate spikes (Figure 6B).

To assess the correlation of LFP among different tetrodes, we calculated the cross correlation. The raw LFP data were firstly bandpass-filtered by delta, theta, alpha, and beta bands. The lag value at maximum cross correlation between different tetrodes was defined as time lag. Based on the configured distance of the tetrodes (Figure 2A insert), the cross-correlation coefficient and time lag were grouped into three groups for comparison.

Sleep states were defined based on EEG–EMG signals and behavioral videos (Liang et al., 2023). Wakefulness was determined by low-amplitude EEG activity and high EMG activity. Non-rapid-eye movement (NREM) sleep was determined by high-amplitude and low-frequency EEG activity, accompanied by low-amplitude EMG activity. Rapid-eye movement (REM) sleep was defined by low-amplitude and high-frequency EEG activity, with no tension in EMG activity. Arousal, NREM sleep, and REM sleep episode durations were manually summarized.

#### 2.7.2 Single-unit activity processing

The raw extracellular electrophysiological data underwent preprocessing to extract spikes, as described previously (Qin et al., 2022). The events detected by each tetrode were initially classified into 4 clusters via a Matlab program, with each cluster representing a distinct wire. Each cluster contained events detected on the corresponding wire, along with those events that also detected by other wires but exhibited the maximum amplitude on this wire. In the high-dimensional feature space, spikes from the same neuron formed clusters, which could be separated from other clusters representing simultaneously recorded cellular and noise events. The MClust toolbox (Schmitzer-Torbert et al., 2005) was employed for sorting these clusters for each unit according to the waveform features such as energy, peak, peak index, valley, valley index, principal component. For the analysis of SUA, neurons were categorized based on the average firing rates recorded during different anesthesia stages, as described earlier.

Putative excitatory neurons and putative interneurons were classified according to spike shape and average firing rate. The trough-peak widths (mean ± S.E.M.) of putative excitatory neurons were 340 ± 4 μs in CA1, and 337 ± 8 μs in DG. The average firing rates (during sleep and wakefulness without isoflurane) of putative excitatory neurons were 2.9 ± 0.2 Hz in CA1, and 3.0 ± 0.2 Hz in DG. Putative interneurons had trough-peak widths of <300 μs, and average firing rates of >12 Hz in both CA1 and DG.

**Figure 7:**
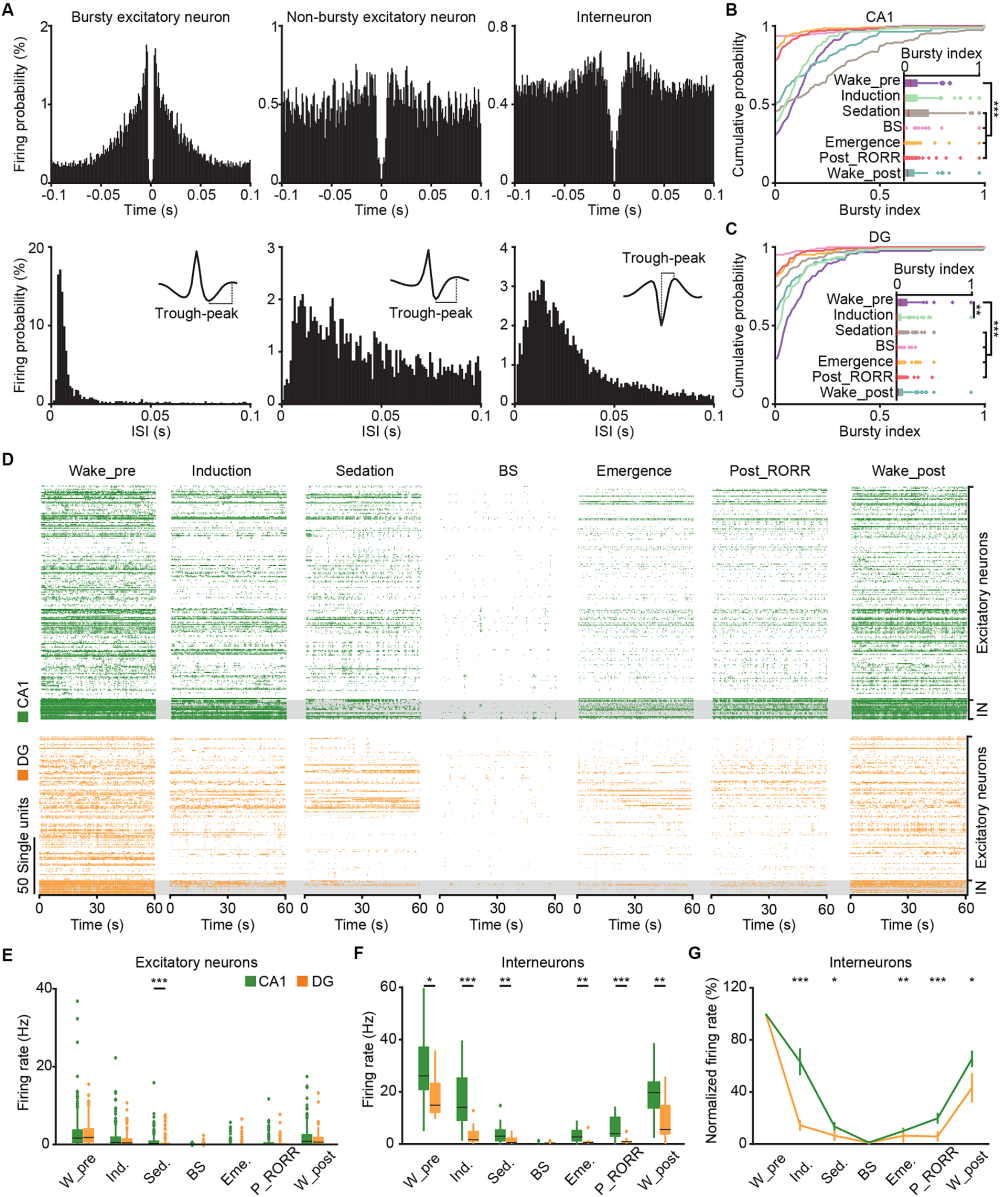
SUA in CA1 and DG in different anesthesia stages. **(A)** Representative spiking-time autocorrelogram (top), ISI hisogram (bottom), and waveform (insert) for bursty excitatory neuron (left), non-bursty excitatory neuron (middle) and interneuron (right). **(B,C)** Cumulative distribution and comparison of the bursty index of excitatory neurons in different anesthesia stages in CA1 (**B**, *n* = 189) and DG (**C**, *n* = 125). Friedman’s ANOVA with Bonferroni post-hoc comparisons test. **(D)** Raster plots showing the SUA of putative excitatory neurons (top) and putative interneurons (bottom) in CA1 (green) and DG (orange) in different anesthesia stages. **(E)** Average firing rates of putative excitatory neurons in CA1 (*n* = 189) and DG (*n* = 125) during different anesthesia stages. **(F)** Average firing rates of putative interneurons in CA1 (*n* = 17) and DG (*n* = 13) during different anesthesia stages. Friedman’s ANOVA with Bonferroni post-hoc comparisons test. Box-and-whisker plot: center line, median; box, 25–75% interquartile range (IQR); the whiskers extend to the most extreme data points that are not outliers. **(G)** The normalized firing rates of CA1 and DG interneurons during different anesthesia stages. Wilcoxon rank-sum test, data are presented as the mean ± SEM. *p < 0.05; **p < 0.01; ***p < 0.001. SUA, single-unit activity. W_pre, wake_pre; Ind., induction; Sed., sedation; Eme., emergence; RORR, recovery of the righting reflex; P_RORR, post_RORR; W_post, wake_post; ISI, interspike interval.

Bursty properties of putative excitatory neurons were evaluated by analyzing the ISI of each unit (Figure 7A). Spike events occurring within a time interval of 3–10 ms ISI were categorized as burst events. A bursty index was calculated as the ratio of burst events to the total number of spikes for quantification.

**Figure 8:**
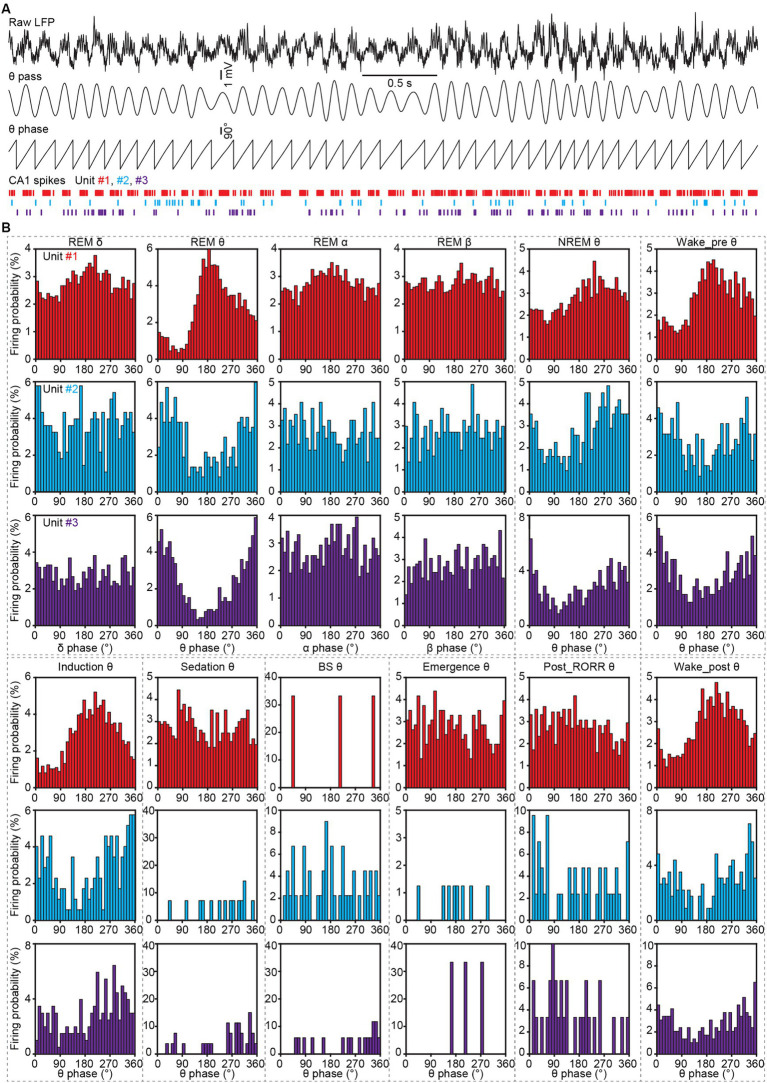
Spike phase locking analysis to LFP oscillations in CA1 during sleep and anesthesia. **(A)** Representative LFP traces (the first trace, raw LFP; the second trace, theta-filtered LFP), theta phase obtained by Hilbert transform, and spike raster of three CA1 neurons. **(B)** Firing histograms of three CA1 neurons (shown in **A**) in different oscillations during different sleep and anesthesia stages. RORR, recovery of the righting reflex.

To analyze the firing phase in delta, theta, alpha and beta oscillations, the raw LFP traces were firstly bandpass-filtered with delta, theta, alpha, and beta bands. Then the angles between the real and imaginary components of the Hilbert transform of the filtered LFPs were defined as the oscillation phase of each band. Subsequently, the oscillation phase was counted at each spike event time during different natural sleep and anesthesia stages (Figure 8).

#### 2.7.3 Statistical analysis

**Figure 9:**
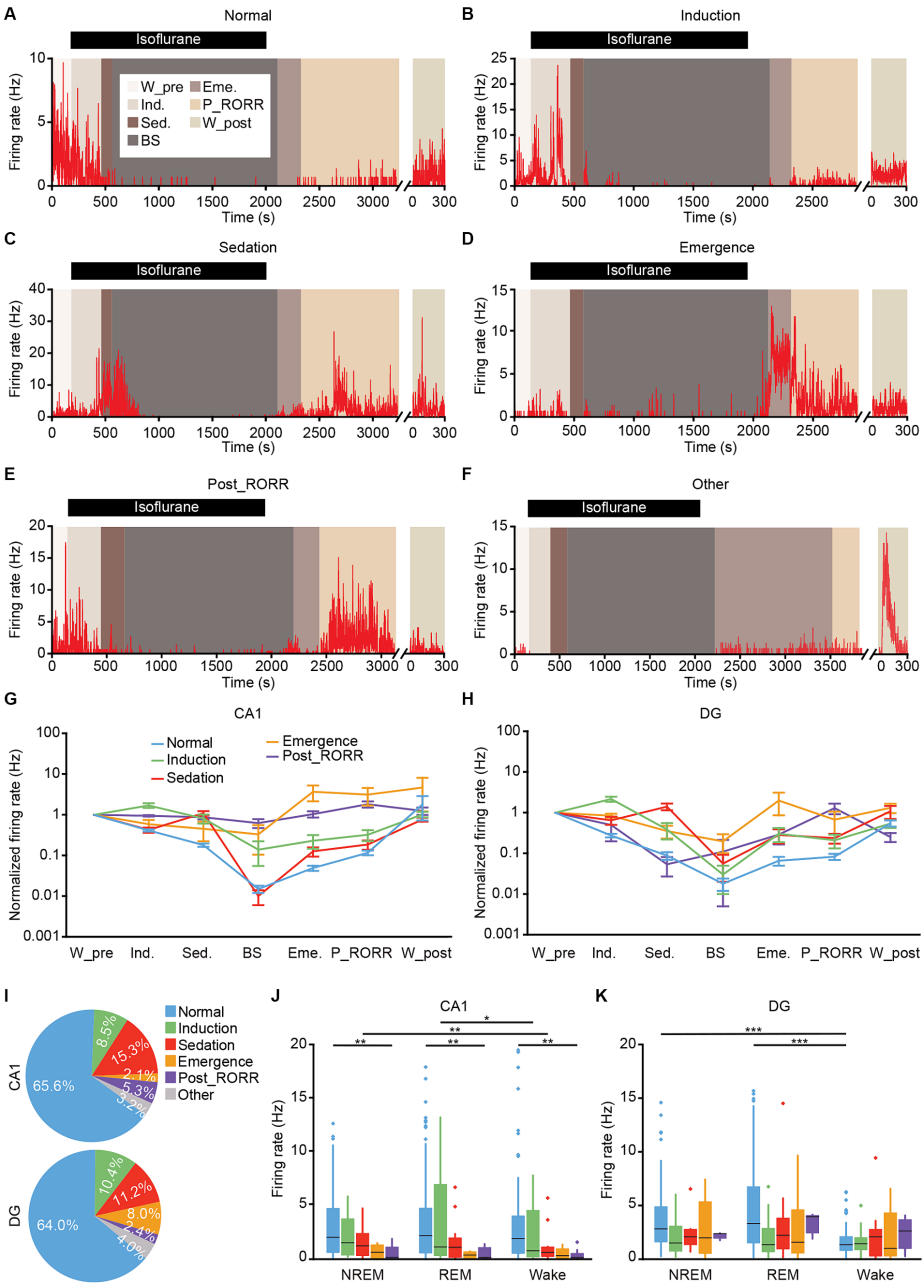
Classification of putative excitatory neurons in CA1 and DG based on the firing rates patterns. **(A–F)** Representative firing rates of different types of DG neurons in different anesthesia stages. Normal type **(A)**; induction type **(B)**; sedation type **(C)**; emergence type **(D)**; post_RORR type **(E)**; and other type **(F)**. **(G,H)** Normalized firing rates of different types of neurons at each stage in CA1 **(G)** and DG **(H)**. Data are presented as the mean ± SEM. **(I)** Percentage of each neuronal type in CA1 (189 neurons) and DG (125 neurons). **(J,K)** Average firing rates of different types of neurons during sleep-wakefulness cycles in CA1 **(J)** and DG **(K)**. CA1, *n* = 189; DG, *n* = 125, Kruskal-Wallis 1-way ANOVA with Tukey post-hoc comparison test. Box-and-whisker plot: center line, median; box, 25–75% interquartile range (IQR); the whiskers extend to the most extreme data points that are not outliers. *p < 0.05; **p < 0.01; ***p < 0.001. RORR, recovery of the righting reflex. W_pre, wake_pre; Ind., induction; Sed., sedation; Eme., emergence; RORR, recovery of the righting reflex; P_RORR, post_RORR; W_post, wake_post.

All statistical analyses were performed using SPSS software. Parametric tests were employed if the data passed both the Shapiro–Wilk normality test and the equal variance test between groups; otherwise, nonparametric tests were used. Student’s *t*-test and the Wilcoxon rank-sum test were utilized for the comparison of two groups (Figures 3B–D,H, 4B–H, 7E–G). One-way ANOVA with LSD *post hoc* comparison and Kruskal-Wallis 1-way ANOVA with Tukey *post hoc* comparison were applied for tests among three or more groups (Figures 2C–F, 9J,K). Repeated measurement data were evaluated by RMs one-way ANOVA with Sidak *post hoc* comparison, and the corresponding nonparametric test was Friedman’s ANOVA with Bonferroni *post hoc* comparison (Figures 3E,F,H, 4I,J, 7B,C,E,F, 9J,K). All tests were two-tailed. The possibility of Type-I errors was kept at 0.05 level for the statistical test conducted only once. When conducting multiple comparisons, we controlled the increased risk of Type-I errors by making LSD, Tukey, Sidak or Bonferroni adjustments to alpha level. For all the boxplots in the figures, the center line represents median, the box represents 25–75% interquartile range (IQR), the whiskers extend to the most extreme data points that are not outliers, and the plus symbols represent outliers (values outside 1.5 * IQR above the upper quartile and below the lower quartile).

## 3 Results

### 3.1 Tetrodes implantation into CA1 and DG

We implanted self-made tetrodes into the CA1 and DG regions to record neuronal activities (Figures 1A,D). Post-recording, tetrode locations were confirmed through histological images (Figures 1B,E). The white traces induced by electrical lesions (red triangles in Figures 1B,E) verified the tetrode tips’ placement in the CA1 pyramidal cell layer and the DG granule cell layer. The positions of all tips of the tetrodes were reconstructed in the mouse brain atlas. The recording sites for CA1 were mainly in the anterior intermediate part of the dorsal CA1 pyramidal layer (Figure 1C). For DG recordings, we had 28 tetrodes in the suprapyramidal region, 16 tetrodes in the infrapyramidal region, and the remaining 4 in the hillus (Figure 1F).

To assess the recordings in hippocampus, we made a comparison of the signals recorded from different tetrodes in freely behaving mice (Figure 2A). The cross correlations and time lags, obtained through lagged cross correlation analysis (Figure 2B), between different tetrodes were used for quantitative comparison. The low-frequency oscillations, including delta, theta, alpha and beta bands, were compared separately. We found that the cross correlation coefficient decreased with the increase of tetrode distance in both CA1(Figure 2C, Kruskal-Wallis test; delta, *H* = 17.0, *p* < 0.001; theta, *H* = 12.2, *p* = 0.002; alpha, *H* = 14.5, *p* < 0.001; beta, *H* = 15.6, *p* < 0.001) and DG (Figure 2E, Kruskal-Wallis test; delta, *H* = 7.2, *p* = 0.03; theta, *H* = 12.0, *p* = 0.002; alpha, *H* = 19.3, *p* < 0.001; beta, *H* = 22.1, *p* < 0.001). Conversely, the time lag increased with the increase of tetrode distance in theta, alpha and beta bands in both CA1 (Figure 2D, Kruskal-Wallis test; theta, *H* = 7.5, *p* = 0.02; alpha, *H* = 9.8, *p* = 0.007; beta, *H* = 9.7, *p* = 0.008) and DG (Figure 2F, Kruskal-Wallis test; theta, *H* = 7.8, *p* = 0.02; alpha, *H* = 6.5, *p* = 0.04; beta, *H* = 9.4, *p* = 0.009). These results, which were consistent with previous study (Lubenov and Siapas, 2009), and along with our histological results, indicated a reliable recording in CA1 and DG.

### 3.2 local field potential and SUA recordings of CA1 and DG in natural sleep

Previous studies have shown that general anesthesia can influence memory and cognition (Skvarc et al., 2018; Tasbihgou and Absalom, 2021), while natural sleep, a state of decreased arousal, is essential for memory consolidation (Diekelmann and Born, 2010). To compare the different impacts of natural sleep and anesthesia on CA1 and DG, we initially conducted recordings in both regions during natural sleep. Electrophysiological data were categorized into LFP (low-pass filtered at 250 Hz) and SUA (high-pass filtered at 250 Hz) based on frequency and synchronized with EEG–EMG signals and behavioral videos for analysis (Figure 3A).

We calculated and compared the LFP power spectral densities in CA1 and DG across delta, theta, alpha, and beta bands during NREM sleep, REM sleep, and wakefulness (Figures 3B–D). Distinct differences in LFPs between CA1 and DG were observed throughout the sleep-wakefulness cycle. During NREM sleep, a significant discrepancy was noted in the beta band of normalized LFP power (Figure 3B right; CA1, 0.30 ± 0.005 versus DG, 0.27 ± 0.008; Wilcoxon signed-rank test, *p* = 0.01). Differences in the theta (CA1, 0.41 ± 0.016 versus DG, 0.36 ± 0.016; unpaired *t*-test, *p* = 0.01) and alpha (CA1, 0.08 ± 0.002 versus DG, 0.1 ± 0.004; Wilcoxon signed-rank test, *p* = 0.01) bands were observed during REM sleep (Figure 3C). Additionally, LFP powers in both CA1 and DG were primarily concentrated in the delta band during NREM sleep, dominated in the theta band during REM sleep and increased in the beta band during wakefulness (Figures 3E,F).

Subsequently, we analyzed SUA in CA1 and DG during sleep-wakefulness cycles. In total, we recorded 189 putative excitatory neurons, 17 putative interneurons in CA1, and 125 putative excitatory neurons, 13 putative interneurons in DG (Figure 3G). The firing rates of DG excitatory neurons were significantly higher than those of CA1 excitatory neurons in both NREM sleep (Figure 3H top; DG, 3.18 ± 0.25 Hz; CA1, 2.52 ± 0.20 Hz; Wilcoxon signed-rank, *p* = 0.002) and REM sleep (DG, 3.87 ± 0.35 Hz; CA1, 2.90 ± 0.27 Hz; Wilcoxon signed-rank test, *p* = 0.001), but showed no difference during wakefulness (DG, 2.47 ± 0.26 Hz; CA1, 3.90 ± 0.43 Hz; Wilcoxon signed-rank test, *p* = 0.97). Moreover, the firing rates of both CA1 and DG excitatory neurons had no significant different across the three sleep-wakefulness states (Friedman test; CA1, *χ*^2^ = 5.0, *p* = 0.08; DG, *χ*^2^ = 5.6, *p* = 0.06).

However, the firing rates of CA1 interneurons were higher than those of DG interneurons in wakefulness (Figure 3H bottom; CA1, 42.4 ± 9.1 Hz; DG, 12.3 ± 2.3 Hz; Wilcoxon signed-rank, *p* = 0.001), but not in NREM sleep (CA1, 22.8 ± 3.3 Hz; DG, 16.9 ± 1.5 Hz; Wilcoxon signed-rank, *p* = 0.54) or REM sleep (CA1, 31.9 ± 3.7 Hz; DG, 27.6 ± 2.4 Hz; Wilcoxon signed-rank, *p* = 0.62). And the firing rates of CA1 interneuron were lower in NREM sleep (Friedman test, *χ*^2^ = 8.8, *p* = 0.01; NREM versus Wake, *p* = 0.03, NREM versus REM, *p* = 0.03), while the DG interneurons showed an increase in firing rates in REM sleep (Friedman test, *χ*^2^ = 17.2, *p* < 0.001; REM versus Wake, *p* < 0.001, REM versus NREM, *p* = 0.005).

### 3.3 Local field potential activities in CA1 and DG under isoflurane-induced general anesthesia

To examine the activities of CA1 and DG during anesthesia, we conducted tetrode recordings in mice exposed to isoflurane-induced general anesthesia (Figure 4A). The peak frequency of the theta band shifted to a lower frequency during the induction stage compared to wakefulness (Figures 4B,C; CA1, 6.21 ± 0.08 Hz versus 8.12 ± 0.09 Hz, *p* < 0.001; DG, 5.51 ± 0.05 Hz versus 7.62 ± 0.03 Hz; paired *t*-test, *p* < 0.001, see stage definition in Methods). Furthermore, the LFP theta activities reduced further after the mice lost their righting reflex in subsequent sedation, BS, and emergence stages (Figures 4D–F). During the recovery from anesthesia, the peak frequencies of LFP delta activities in CA1 initially increased after the mice regained their righting reflex (Figures 4F,G; emergence stage, 2.16 ± 0.03 Hz versus post_RORR stage, 2.69 ± 0.06 Hz; paired *t*-test, *p* < 0.001). Subsequently, the LFP theta activities gradually recovered when mice resumed locomotion in the wake_post stage (Figure 4H).

We compared the differences in LFP power between CA1 and DG across different anesthesia stages. In the induction stage, the normalized power of the theta band in CA1 was stronger than that in DG (Figure 4C; CA1, 0.26 ± 0.010 versus DG, 0.24 ± 0.008; unpaired *t*-test, *p* = 0.01). No significant differences in normalized LFP powers were observed between CA1 and DG in sedation and BS stages (Figures 4D,E). However, significant differences in the delta band (Figure 4F; CA1, 0.24 ± 0.02 versus DG, 0.19 ± 0.01; Wilcoxon signed-rank test, *p* = 0.04) of the emergence stage and in the alpha band (Figure 4G; CA1, 0.09 ± 0.002 versus DG, 0.10 ± 0.002; unpaired *t*-test, *p* = 0.03) of the post_RORR stage were observed. After the mice recovered from anesthesia in the wake_post stage, CA1 LFP exhibited higher theta power (CA1, 0.28 ± 0.01 versus DG, 0.24 ± 0.01; unpaired *t*-test, *p* = 0.01) and lower delta power (CA1, 0.17 ± 0.01 Hz versus DG, 0.21 ± 0.01 Hz; unpaired *t*-test, *p* = 0.01) than DG LFP.

We then compared the changes in normalized power in different LFP bands throughout the entire isoflurane-induced general anesthesia process (Figures 4I,J). For the delta band in both CA1 and DG LFPs, the power gradually increased and maximized in the BS stage. Subsequently, it started to decrease after isoflurane was turned off and gradually recovered to the pre-anesthesia level (Friedman test; CA1, *χ*^2^ = 31.6, *p* < 0.001; DG, *χ*^2^ = 49.9, *p* < 0.001). Regarding the theta band, the normalized power began to decrease after isoflurane delivery and remained low in the subsequent sedation, BS, emergence, and post_RORR stages in both CA1 (Friedman test, *χ*^2^ = 39.4, *p* < 0.001) and DG (Friedman test, *χ*^2^ = 35.6, *p* < 0.001). However, CA1 theta power recovered faster than DG. For the alpha and beta bands, which has been reported to originate from the somatostatin neurons in amygdala (Jackson et al., 2024), their normalized power decreased only in the BS stage in both CA1 and DG.

Previous study has suggested that hippocampal LFP phase was modulated to slow-wave activity during anesthesia (Yang et al., 2021). We then examined the cross-frequency coupling between the phase of slow LFP oscillations and the amplitude of gamma oscillation across different sleep and anesthesia states by doing phase-amplitude coupling (PAC, Figure 5A). During REM sleep, a clear coupling between theta and gamma bands was found, while an additional coupling between delta and gamma bands was observed during NREM sleep (Figure 5B). After isoflurane delivery, the coupling phase shifted from theta to lower frequency gradually (Figure 5B wake_pre, induction and sedation). And after isoflurane shut off, the coupling phase increased from delta band into theta band (Figure 5B emergence, post_RORR and wake_post).

Sharp-wave ripple and dentate spike, which happen in CA1 and DG, respectively, during awake immobility and NREM sleep, are two typical hippocampal LFP activities (Farrell et al., 2024). We additionally checked these two activities in our datasets. We found that both ripples and dentate spikes occurred frequently during NREM sleep, disappeared with isoflurane delivery, and were regenerated after recovery from anesthesia (Figures 6A,B).

### 3.4 Single-unit activity of CA1 and DG neurons during isoflurane-induced general anesthesia

We conducted an analysis to characterize the spike firing patterns of CA1 and DG neurons at the single-cell resolution throughout general anesthesia by examining the busty index and firing rate. The spike-time autocorrelogram, ISI histogram and waveform of typical bursty, non-bursty excitatory neuron and interneuron were shown in Figure 7A. A total of 206 CA1 neurons (189 putative excitatory neurons, 17 putative interneurons) and 138 DG neurons (125 putative excitatory neurons, 13 putative interneurons) were recorded. The bursty index of both CA1 (Figure 7B, *n* = 189, Friedman test, *χ*^2^ = 317.1, *p* < 0.001) and DG (Figure 7C, *n* = 125, Friedman test, *χ*^2^ = 193.7, *p* < 0.001) excitatory neurons reduced with the isoflurane delivery, and increased with the recovery from anesthesia. The firing sequences of all recorded neurons across anesthesia stages were depicted in Figure 7D. The average firing rates of putative excitatory neurons in both CA1 and DG gradually reduced with the deepening of anesthesia, followed by a gradual recovery after the cessation of isoflurane (Figures 7D,E, Friedman test; CA1, *χ*^2^ = 678, *p* < 0.001; DG, *χ*^2^ = 415; *p* < 0.001). Notably, during the sedation stage, the firing rates of DG excitatory neurons were significantly lower than those of CA1 (Figure 7E, Wilcoxon signed-rank test, *p* < 0.001).

For the interneurons, the firing rates in CA1 and DG also decreased with the application of isoflurane, and increases with the recovery from anesthesia (Figures 7D,F, Friedman test; CA1, *χ*^2^ = 87, *p* < 0.001; DG, *χ*^2^ = 60; *p* < 0.001). And the firing rates of DG interneurons were consistently lower than that in CA1 except for BS stage (Figure 7F, Wilcoxon signed-rank test; wake_pre, *p* = 0.02; induction, *p* < 0.001; sedation, *p* = 0.009; emergence, *p* = 0.001; post_RORR, *p* < 0.001; wake_post, *p* = 0.002). What’s more, the DG interneurons decreased their firing rates faster in the induction and sedation stages of anesthesia (Figure 7G, Wilcoxon signed-rank test; induction, CA1, 63 ± 9.8%, DG, 14 ± 3.7%, *p* < 0.001; sedation, CA1, 13 ± 3.9%, DG, 6 ± 3.5%, *p* = 0.02), and increased slower after the cessation of isoflurane (emergence, CA1, 10 ± 1.9%, DG, 6 ± 4.7%, *p* = 0.006; post_RORR, CA1, 20 ± 3.6%, DG, 6 ± 3.4%, *p* < 0.001; wake_post, CA1, 65 ± 5.7%, DG, 43 ± 10.9%, *p* = 0.02) than CA1 interneurons.

Hippocampal neurons have been reported to be phase-locked to theta oscillation during navigation and memory (Reddy et al., 2021). To test whether the CA1 spikes were phase-locked to LFP oscillations in different sleep and anesthesia stages, we calculated the firing phase of neurons. The firing histograms of an interneuron and two excitatory neurons were shown in Figure 8. We found these three neurons were phase-locked to theta, but not to delta, alpha or beta bands. And this theta phase-locking feature was manifested in REM sleep, NREM sleep, wake_pre, induction and wake_post stages, with the preferred firing phase remaining stable.

While the general trend for CA1 and DG neurons involved a decrease in firing rates during isoflurane delivery and an increase after its removal, individual neurons exhibited distinct firing patterns. We identified six neuronal types based on their firing rates across different anesthesia stages. The normal-type, comprising the largest neuron population, exhibited a gradual decrease in firing rate with isoflurane administration and an increase after its removal (see example in Figure 9A, summary in Figures 9G,H blue line). The induction-type displayed a higher firing rate during the induction stage (see example in Figure 9B, summary in Figures 9G,H green line). Three other types of neurons, showing high firing rates during the sedation, emergence, or post_RORR stage, were categorized as sedation-type (see example in Figure 9C, summary in Figures 9G,H red line), emergence-type (see example in Figure 9D, summary in Figures 9G,H orange line), and post_RORR-type (see example in Figure 9E, summary in Figures 9G,H purple line). The remaining neurons with irregular firing patterns were grouped as other-type (see example in Figure 9F).

We then summarized the proportion of each neuronal type in CA1 and DG, respectively (Figure 9I). Almost all the interneurons belonged to normal-type (CA1, 16 of 17 units, DG, 13 of 13 units). For the excitatory neurons, approximately 64–66% of both CA1 and DG neurons were normal-type. The induction-type shared a similar proportion in both regions. However, the sedation-type (CA1, 15.3%; DG, 11.2%) and post_RORR-type (CA1, 5.3%; DG, 2.4%) occupied a larger proportion in CA1 than in DG. Conversely, the proportion of emergence-type in DG (8.0%) was larger than in CA1 (2.1%). We further compared the firing rates of the five neuronal types for excitatory neurons during sleep-wakefulness cycles. In the CA1 region, the firing rates during NREM sleep were higher than during wakefulness for the sedation-type (Figure 9J; NREM, 1.75 ± 0.24 Hz, wake, 1.48 ± 0.28 Hz, Friedman test, *p* = 0.002). Additionally, the firing rates of the induction-type during REM sleep were significantly higher than during wakefulness (Figure 9J; REM, 3.82 ± 1.17 Hz, wake, 2.35 ± 0.76 Hz; Friedman test, *p* = 0.02). Furthermore, a significant difference in firing rate across the sleep-wakefulness cycle was only observed between normal-type and post_RORR-type (Kruskal-Wallis test; NREM, *p* = 0.002, REM, *p* = 0.003, wake, *p* = 0.001). For the DG region, only normal-type neurons showed lower firing rates during wakefulness than during NREM sleep and REM sleep (Figure 9J; NREM, 3.73 ± 0.35 Hz, REM, 4.67 ± 0.47 Hz, wake, 1.65 ± 0.16 Hz; Friedman test; NREM versus wake, *p* < 0.001, REM versus wake, *p* < 0.001).

## 4 Discussion

CA1 and DG exhibit distinct structures and functions (Alkadhi, 2019). However, the varied effects of isoflurane-induced general anesthesia on neuronal activities in CA1 and DG remain unexplored. In this study, employing tetrode recordings, we observed LFP and SUA in both CA1 and DG throughout natural sleep and the general anesthesia process. LFP activities displayed parallel changes between CA1 and DG during natural sleep and general anesthesia, with the exception of earlier recovery of theta activity in CA1 compared to DG (Figure 4). Additionally, at the single-cell level, we noted a higher firing rate in DG excitatory neurons compared to CA1 during NREM and REM sleep (Figure 3H), but a lower firing rate in the sedation stage (Figure 7E). We also discovered that the firing rates of DG interneurons reduced more rapidly than those of CA1 interneurons in the early period of anesthesia and recovered slower during the recovery phase. Furthermore, neurons recorded during general anesthesia were classified based on firing rate patterns, revealing higher proportions of sedation-type and post_RORR-type neurons in CA1, while a higher proportion of emergence-type neurons in DG (Figure 9I).

The features of LFP in hippocampal CA1 and DG during natural sleep in our study align with previous findings (Herweg et al., 2020; Girardeau and Lopes-Dos-Santos, 2021). Delta waves, a characteristic electrophysiological pattern during NREM sleep, associated with the down states of classical slow oscillation (Girardeau and Lopes-Dos-Santos, 2021), play a role in memory consolidation by coordinating with other hippocampal and cortical regions (Maingret et al., 2016). For REM sleep, theta waves were predominant LFP patterns in hippocampal regions involved in memory consolidation (Boyce et al., 2016; Herweg et al., 2020; Qin et al., 2022). In contrast, general anesthesia induced memory and cognition impairments (Skvarc et al., 2018; Tasbihgou and Absalom, 2021). In this study, the most notable changes during anesthesia were the increase in delta power and the decreases in theta, alpha, and beta powers, consistent with previous studies in CA1 (Yang et al., 2021). However, our precise description of the anesthesia process revealed that the increase in delta power was primarily in the BS stage. The decrease in theta band mainly occurred after mice lost their righting reflex, while the decreases in alpha and beta powers were only in the BS stage (Figures 4I,J). Delta waves and theta rhythms are considered indicators of levels of consciousness (Ma and Leung, 2006). Although general anesthesia and sleep share similarities in decreased arousal state, LFP changes under anesthesia differ from natural sleep.

At the single-cell level, the firing rates of most CA1 and DG neurons significantly decreased after isoflurane administration and gradually increased after recovery from anesthesia. Isoflurane has been reported to reduce pyramidal cell activity by increasing GABAergic synaptic inhibition (Nishikawa and MacIver, 2001) or inhibiting sodium currents (Zhao et al., 2019). It has been also shown that interneurons are capable of modulating neuronal activity and may be involved in the neural activity modifications by isoflurane anesthesia (Jha and Mallick, 2009; Wang Y. et al., 2023). However, the firing rates of both CA1 and DG neurons were not reduced after mice got into natural sleep. In summary, isoflurane robustly reduced neuronal activities in both CA1 and DG, differing from natural sleep, and may lead to cognitive impairments. Notably, a small population of neurons increased their firing rates during different stages of general anesthesia.

During REM sleep, we observed a higher theta power in CA1 compared to the dentate gyrus (DG), consistent with previous investigations (Buzsáki, 2002; Qin et al., 2022). Furthermore, our findings indicate an earlier recovery of theta rhythm in CA1 than in DG under anesthesia. This disparity may be attributed to the involvement of pyramidal cells in CA3 and entorhinal cortex layer III in theta generation (Buzsáki, 2002; Suh et al., 2011).

The lower firing rates observed in DG excitatory neurons during sedation stage as well as in DG interneurons throughout the entire anesthesia process except for BS stage, suggest a greater sensitivity and prolonged impact of isoflurane on DG. Previous studies have demonstrated that isoflurane enhances synaptic GABA-A inhibition and depresses glutamate NMDA receptor function (Bieda et al., 2009; Wang et al., 2020). Notably, GABA-A receptors play a crucial role in both synaptic and tonic inhibition, with stronger implications in DG than in CA1 (Arima-Yoshida et al., 2011). Additionally, the expression of the NMDA receptor GluN1 subunit is higher in DG compared to CA1 (Coultrap et al., 2005). Therefore, the diverse effects of isoflurane-induced general anesthesia on hippocampal subregions may be attributed to variations in the expression levels of neuronal receptors, potentially leading to distinct cognitive impairments (Engin et al., 2015; Jovasevic et al., 2015).

Taken together, our discoveries contribute to the direct measurements of neuronal activities across anesthesia in hippocampal subregions. The mechanisms underlying the distinct activity patterns between DG and CA1 are worthy of further exploration by applying multiple isoflurane concentrations and combining with behavioral correlates. Anesthesia can induce both cognitive disorders and sleep disturbances concurrently or independently (Bilotta et al., 2016; Kumar and Jha, 2017; Luo et al., 2020), and also influence the maintenance of gravity and posture (Gonfalone and Jha, 2015). These anesthesia-induced behavioral disorders need to be comprehensively considered and systematically studied in the future.

## Data availability statement

The original contributions presented in the study are included in the article/supplementary material, further inquiries can be directed to the corresponding author.

## Ethics statement

The animal study was approved by Third Military Medical University Animal Care and Use Committee. The study was conducted in accordance with the local legislation and institutional requirements.

## Author contributions

RW: Writing – review & editing, Writing – original draft, Visualization, Validation, Software, Resources, Project administration, Investigation, Formal analysis, Conceptualization. LZ: Writing – review & editing, Writing – original draft, Visualization, Supervision, Software, Methodology, Funding acquisition, Formal analysis, Data curation, Conceptualization. XiaW: Writing – review & editing, Validation, Methodology, Investigation, Formal analysis, Data curation. WL: Writing – review & editing, Visualization, Validation, Methodology, Investigation, Formal analysis, Data curation, Conceptualization. TJ: Writing – review & editing, Visualization, Supervision, Methodology, Investigation, Funding acquisition, Conceptualization. PY: Writing – review & editing, Validation, Methodology, Investigation, Formal analysis, Data curation. XinW: Writing – review & editing, Validation, Methodology, Investigation, Formal analysis, Data curation. QC: Writing – review & editing, Validation, Methodology, Investigation, Formal analysis, Data curation. XC: Writing – review & editing, Supervision, Software, Methodology, Funding acquisition, Data curation. HQ: Writing – original draft, Writing – review & editing, Visualization, Supervision, Software, Project administration, Methodology, Investigation, Funding acquisition, Formal analysis, Data curation, Conceptualization.
